# Lack of placental neurosteroid alters cortical development and female somatosensory function

**DOI:** 10.3389/fendo.2022.972033

**Published:** 2022-10-13

**Authors:** Dana Bakalar, Jiaqi J. O’Reilly, Helene Lacaille, Jacquelyn Salzbank, Jacob Ellegood, Jason P. Lerch, Toru Sasaki, Yuka Imamura, Kazue Hashimoto-Torii, Claire-Marie Vacher, Anna A. Penn

**Affiliations:** ^1^ Center for Neuroscience Research, Children’s Research Institute, Children’s National Health System, Washington, DC, United States; ^2^ Division of Neonatology, Department of Pediatrics, NewYork-Presbyterian Morgan Stanley Children’s Hospital, Vagelos College of Physicians and Surgeons, Columbia University, New York, NY, United States; ^3^ Mouse Imaging Centre (MICe), Hospital for Sick Children, Toronto, ON, Canada; ^4^ Wellcome Centre for Integrative Neuroimaging (WIN), Nuffield Department of Clinical Neurosciences, John Radcliffe Hospital, University of Oxford, Oxford, United Kingdom; ^5^ Department of Biochemistry and Molecular Biology, Pennsylvania State University College of Medicine, Hershey, PA, United States

**Keywords:** neuroplacentology, allopregnanolone (3α,5α-THP), somatosensory cortex (S1), placenta, postmortem human brain, preterm birth, GABAA receptor (GABAAR)

## Abstract

Placental endocrine function is essential to fetal brain development. Placental hormones include neurosteroids such as allopregnanolone (ALLO), a regulator of neurodevelopmental processes *via* positive allosteric modulation of the GABA_A_ receptor (GABA_A_-R). Using a mouse model (plKO) in which the gene encoding the ALLO synthesis enzyme is specifically deleted in trophoblasts, we previously showed that placental ALLO insufficiency alters cerebellar white matter development and leads to male-specific autistic-like behavior. We now demonstrate that the lack of placental ALLO causes female-predominant alterations of cortical development and function. Placental ALLO insufficiency disrupts cell proliferation in the primary somatosensory cortex (S1) in a sex-linked manner. Early changes are seen in plKO embryos of both sexes, but persist primarily in female offspring after birth. Adolescent plKO females show significant reduction in pyramidal neuron density, as well as somatosensory behavioral deficits as compared with plKO males and control littermates. Assessment of layer-specific markers in human postmortem cortices suggests that preterm infants may also have female-biased abnormalities in cortical layer specification as compared with term infants. This study establishes a novel and fundamental link between placental function and sex-linked long-term neurological outcomes, emphasizing the importance of the growing field of neuroplacentology.

## Introduction

A key role of placental hormones in neurodevelopment has long been hypothesized (1–3). Placental dysfunction (e.g. due to infection or malperfusion) and premature loss of the placenta (i.e. preterm birth) are associated with abnormal brain development and neurological and cognitive deficits, even in late-preterm infants who are not otherwise ill (2, 4–9). One important placental hormone is allopregnanolone (3α,5α-tetrahydroprogesterone or ALLO), which derives from progesterone *via* a two-step enzymatic pathway concluding with the action of 3α-hydroxysteroid dehydrogenase (3α-HSD), an enzyme encoded by the *akr1c14* gene in mice. ALLO levels increase during mid-to-late pregnancy in both mother and fetus (10, 11), and although the brain can produce ALLO from its precursors starting near term (12–14), fetal ALLO is primarily placental in origin (15).

ALLO is a potent allosteric modulator of the ligand-gated GABA_A_ receptor (GABA_A_-R), a receptor involved in the regulation of many neurodevelopmental processes such as neurogenesis, cell survival, cell migration and synaptogenesis (16–22). ALLO has been shown to control cell proliferation in multiple model systems and cell types *via* its action on GABA_A_-Rs (23). Treatment with ALLO acts on intermediate progenitor cells (IPCs), increasing proliferation in the cortical subventricular zone (SVZ) and down-regulating genes that promote cell cycle exit (22, 24, 25).

Fetal brain ALLO levels peak in mid-to-late gestation (15, 26, 27), which coincides with the formation and expansion of the cerebral cortex. In mouse, placental expression of the *akr1c14* gene is highest at about embryonic day (E) 15 (15), a stage at which the number of IPCs quickly expands in the SVZ (28, 29). IPCs give rise to post-mitotic newborn excitatory pyramidal neurons that will migrate to the upper layers (layers II-IV, or ULs) of the developing cortex (30). IPCs express GABA_A_-Rs, enabling GABAergic regulation over their proliferation, differentiation and the migration of their daughter cells (31), further suggesting a role for ALLO during development. Human studies reported a similar rise in fetal ALLO during mid-to-late gestation (32, 33), coinciding with active IPC production in the SVZ (34). In humans, the ratio of UL versus deeper layer (DL) neurons is extraordinarily high, and likely contributes to cortical gyrification (35, 36), suggesting that loss of ALLO may cause significant damage to human neurodevelopment. In the present study, we have tested the hypothesis that insufficient placental ALLO affects cerebral cortex development in mouse and human. Loss of placental ALLO in preterm infants during this critical window of corticogenesis is a potential devastating factor for the developing brain, contributing to the etiology of prematurity cognitive disorders.

Much of previous research relied on pharmacologically manipulating ALLO levels, which differs from directly altering *de novo* ALLO during development. ALLO’s actions at GABA_A_-Rs are concentration-dependent (37), so supplementing ALLO can change its pharmacodynamics and alter downstream signaling pathways (18, 38–41). Studies blocking ALLO *via* pharmacological agents present compelling findings for a role of ALLO during development, but this approach alters a wide class of other hormones (42–44). Importantly, these pharmacological approaches could not specifically assess the *placental* contribution of ALLO to fetal neurodevelopment. The Cre-Lox akr1c14^Cyp19^ KO (plKO) mouse model that we recently developed overcomes these limitations by its tissue specificity, enabling us to isolate the consequences of placental ALLO insufficiency on brain development. Using this model, we have previously found that placental ALLO supports cerebellar white matter development, and that its depletion increases the risk of Autism Spectrum Disorders (ASD)-like features in male, but not female, progeny (15). Since much of corticogenesis occurs embryonically, when fetal (placental) ALLO is high, we aimed to determine if placental ALLO contributes to the development of the cortex.

Here we demonstrate in the plKO mouse model that reduced placental ALLO production results in predominantly female impairments of cortical development, in particular in the primary somatosensory cortex (S1). Furthermore, the analysis of human postmortem cortical specimens suggests that preterm birth causes similar sex-biased disruption in the upper-layer formation. Overall, our study shows that placental hormones such as ALLO support fetal brain development in a sex- and region-dependent manner, and that their loss may contribute to long-term neurobehavioral outcomes of prematurity.

## Materials and methods

### Mice

#### Animal care and use accreditations

Animals were group housed with a 12 hour light/dark cycle and ad libidum access to food and water. Mice were bred and maintained at the Association for Assessment and Accreditation of Laboratory Animal Care International accredited Children’s National Research Institute Animal Facility. All experiments and procedures were done in compliance with the Animal Welfare Act, the regulatory requirements of the NIH Office of Laboratory Animal Welfare and the US Department of Agriculture and were approved by the Institutional Animal Care and Use Committee [protocol no. 30534 (PI: Penn)]. Children’s National Research Institute has currently approved Animal Welfare Assurance Agreements with the PHS Office of Laboratory Animal Welfare [D16-00219 (A3338-01)].

#### Akr1c14-floxed mice

The akr1c14-floxed mouse (akr1c14^fl/fl^) was created by Ingenious Targeting Laboratories (Ronkonkoma, NY, USA), as previously described (15).

#### Cyp19-Cre mice

The Cyp19-Cre mouse line (#5912 line), which expresses Cre exclusively in trophoblast cells, was obtained from Dr. Gustavo Leone, Ohio State University (45).

#### Akr1c14^Cyp19^ (plKO) mice

The akr1c14^Cyp19a^KO (plKO) mouse was generated by successive crosses from progenitor akr1c14-floxed and Cyp19-Cre animals. Homozygous Cyp19-Cre females were crossed to homozygous akr1c14-floxed males. Resulting females (hemizygous for Cre and heterozygous for LoxP sites flanking *akr1c14*) were mated to homozygous akr1c14-floxed males. Female offspring homozygous for LoxP and positive for Cre were crossed with homozygous akr1c14-floxed males, resulting in experimental animals, the plKO mice and littermate controls (Ctrl). The generation and genetic validation of the plKO mice has been previously described (15). Both male and female offspring were used in all studies described. The body weight was monitored across postnatal development into adulthood for all genotypes.

#### PCR genotyping

Genomic DNA was extracted using the Extracta DNA Prep for PCR kit and genotyped with AccuStart II GelTrack PCR SuperMix (Quanta Biosciences, Gaithersburg, MD, USA). Primers used to genotype and sex animals are available in **Supplementary Table 1**. PCR was run in a 2720 ThermoCycler (Applied Biosystems, Waltham, MA, USA) with the following program: 94° for 3 minutes; (94° for 30 seconds, 60° for 30 seconds, 72° for 1 minute) x 30 cycles; 72° for 10 minutes.

### Human samples

The NIH NeuroBioBank (University of Maryland, Baltimore, MD) provided the frozen human Brodmann Area 10 cortex samples (request ID #709). Donors comprised 6-week-old term infants (average age) and corrected age matched preterm infants. Those with major congenital anomalies or known genetic diagnoses, and those with meningitis or stroke as cause of death were excluded. The sex, completed gestational weeks and cause of death varied, but the majority were caused by sudden infant death syndrome, with no CNS infection, hemorrhage or malformation noted. Donors’ information were previously reported in (46).

### BrdU and EdU labeling of proliferating cells

Proliferating cells were labeled *in utero* (at E15.5, determined by observation of vaginal plugs, the day of plug being E0.5) by maternal intraperitoneal injection of BrdU (50 mg/kg of maternal body weight; Thermo Fisher Scientific, Waltham, MA, USA) or EdU (123 mg/kg; Thermo Fisher Scientific) (47) dissolved in sterile normal saline solution. 10% DMSO was added to dissolve EdU in the saline solution.

### Tissue collection

#### Embryonic brains

Gestational age was determined by observation of vaginal plugs (the day the plug was observed is day 0.5). For collection of embryonic tissue, dams were euthanized using carbon dioxide exposure followed by cervical dislocation. Embryos were then dissected out and decapitated. Brains were removed from the skull and either post-fixed in 4% paraformaldehyde (PFA) for 1-2 days, then transferred to 30% sucrose at 4° until equilibrium (for immunohistochemistry), or flash frozen in liquid nitrogen and stored at -80° until use (for Western blots).

#### Postnatal brains

At P30, animals were euthanized either *via* carbon dioxide asphyxiation or by transcardial perfusion with 4% PFA under deep isofluorane anesthesia. For RNA extractions, cerebral cortex was dissected and flash frozen with liquid nitrogen and stored at -80°C until use. For immunohistochemistry, brains were post-fixed overnight in 4% PFA at 4°C. All fixed tissues underwent a sucrose gradient of 10%, 20% and 30% over three days at 4°C.

### 3’mRNA-sequencing

#### Tissue collection and RNA extraction

Bilateral cerebral cortex from Ctrl and plKO mice (males and females; 3 animals/group) were dissected at P30 and flash frozen. Tissue homogenization and total RNA extraction were performed using the *mir*Vana Isolation kit (Thermo Fisher Scientific; #AM1560) as per the manufacturer’s instructions.

#### Library Preparation, Sequencing for mRNA and Data Analysis

All the procedures have been described previously (15). The data have been deposited in NCBI’s Gene Expression Omnibus and are accessible through GEO Series accession number GSE173440. Principal component analysis and scatter matrices revealed no outliers in our biological replicates. Differentially expressed genes had to meet the following criteria: p<0.05; fragments per kb of transcript per million mapped reads values more than one; and fold change greater than 1.5. These cutoffs’ limits were validated using qRT-PCR on cortical samples from both similar and different mouse cohorts. Ingenuity Pathway Analysis (Qiagen, Redwood City, CA, USA) was used to identify the top differentially regulated biological functions and disease processes.

### qRT-PCR

Mouse S1 somatosensory cortex and human Brodmann Area 10 tissues were homogenized in TRIzol™ Reagent (Thermo Fisher, Waltham, MA, USA; #15596018); total RNA was extracted with the RNeasy Mini Kit (Qiagen Venlo, The Netherlands; #74104) and quantified with a Nanodrop ND-2000C (Thermo Fisher). 1 μg of RNA was used to make cDNA with the iScript cDNA Synthesis Kit (Bio-Rad, Hercules, CA, USA; #1708891). All primer pairs were designed and validated in-house for efficiency and specificity. RT-PCR experiments were performed on cDNA samples in presence of SsoAdvanced Universal SYBR Green Supermix (Bio-Rad; #1725271) with specific primers at 100 nM using the ABI Prism 7500 Sequence Detection System (Thermo Fisher). The cDNA-generated signals for target genes were normalized with the glyceraldehyde 3-phosphate dehydrogenase (*gapdh*). Primers used for qRT-PCR are available in **Supplementary Table 1**. The regulation was determined with the 2^–ΔΔCq^ method. Results are expressed as fold change compared to the control group.

### Western blots

Protein extracts from E15.5 female cortices were harvested by sonication in 1× RIPA lysis buffer and quantified for concentration using the Pierce BCA protein assay (Thermo Fisher Scientific). 25 μg of proteins were separated by SDS-PAGE and transferred onto PVDF membranes (0.2-μm-large pores) using the Bio-Rad system. Blots were blocked for one hour with 5% non-fat dry milk in TBS with 0.1% Tween-20 (TBS-T) and incubated overnight at 4°C in 3% BSA in TBS-T with antibodies against: BCL-2 (1:1,000; Cell Signaling, Danvers, MA, USA; #2772), BAX (1:1,000; Cell Signaling; #2772) or β-tubulin (1:2,000; Cell Signaling; **#** 2146). HRP-conjugated secondary antibodies, mouse IgG kappa binding protein (1:2,000; Santa Cruz; #sc-51602) or anti-rabbit IgG (1:2,000; Cell Signaling; #7074) were incubated for one hour, and chemiluminescence was detected using Clarity Western ECL Substrate on a Bio-Rad ChemiDoc MP imaging system. Using ImageJ software (http://rsb.info.nih.gov/ij/), all targets were analyzed densitometrically relative to β-tubulin loading control, and then were normalized to the average density of all bands within each blot to account for inter-run variability.

### Immunohistochemistry

Brains used for immunohistochemistry were sectioned coronally at 20 µm (embryonic brains) or 40 µm (P30 brains) using a sliding microtome (Thermo Fisher Scientific). EdU incorporation was detected by reaction with azide-conjugated Alexa555, following manufacturer instructions (Click-iT EdU Alexa Fluor 555 Imaging Kit, Thermo Fisher Scientific). Briefly, sections were incubated for 30 min in the dark in freshly made Click-iT reaction cocktail and then washed in PBS containing 0.3% Triton-X (PBST). BrdU, when used, was uncovered by a 30 min NAOH 2N submersion, followed by washing in PBST. Sections were blocked for 1.5 hours at room temperature in PBST containing 10% normal donkey serum and 0.3% Triton X-100, before being incubated overnight at 4° with primary antibodies. The next day, sections were washed 3 times with PBST and incubated in secondary antibodies at room temperature for 1.5 hours, then washed as before and mounted in Fluoromount-G mounting medium (Southern Biotech, Birmingham, AL, USA). Staining was performed using the following primary antibodies: BrdU (1:500; Abcam; #ab6326), COUP-TF-interacting protein 2 (Ctip2; also called BCL11B; 1:500; Novus biologicals, Littlecon, CO, USA; #NB100-2600), Special AT-rich sequence-binding protein 2 (Satb2; 1:250; Abcam; #ab51502), Cut like homeobox 1 (Cux1; also called CDP; 1:1,000; Santa Cruz; #sc-6327), Forkhead box protein P2 (FoxP2; 1:1,000; Abcam; #ab16046), Ki67 (1:250; Abcam; #ab15580) or phospho-Histone 3 (pH3; 1:500; Santa Cruz; #sc-56745). The following secondary antibodies were used at 1:2,000: donkey anti-mouse Alexa-488 (Invitrogen; #A-21202), donkey anti-rabbit Alexa-488 (Invitrogen; #A-21206), donkey anti-mouse Alexa-555 (Invitrogen; #A-31570), donkey anti-rabbit Alexa-555 (Invitrogen; #A-31572), donkey anti-mouse Alexa-647 (Invitrogen; #A-31571), donkey anti-rabbit Alexa-647 (Invitrogen; #A-31573).

### Imaging and quantification

#### Imaging procedures

Brain sections were selected to sample the developing and adult somatosensory cortex. Embryonic sections were selected from the middle of the embryonic brain’s rostral caudal axis [Allen Developing Brain Reference Atlases, TS24 (E15.5) and TS25 (E17.5)]. A minimum of two sections from each animal were analyzed in each staining, and the counts were averaged to provide the final data used. For postnatal brain sections, four images were taken of the barrel subfield of S1 in each hemisphere of each cortex (based on the Allen Brain Reference Atlas), and data from these images was averaged. Confocal images (Z-stacks of between 5 and 8 1-2-μm optical sections) of all sections were acquired with an Olympus FV1000 laser scanning confocal microscope through a 20× oil objective. Four different laser lines were used for imaging: FITC (488 nm excitation; 522/35 emission filter), Cy3 (560 nm excitation; 605/32 emission filter), Cy5 (647 nm excitation; 680/32 emission filter) and DAPI (400 nm excitation). All counting was performed by an individual blind to group allocation of the animals.

#### Cell proliferation assays

Mitosis was examined by staining for pH3 (marking mitotic cells), and the number of pH3+ cells in a 450-µm by 350-µm ROI including the ventricular zone of the cortex was assessed. Imaris-Bitplane software (South Windsor, CT, USA) was used for embryonic cell counts. Cells were identified using the “spots” function. The “colocalize spots” plugin was further used to identify co-localization of antigens. Spot criteria were adjusted until visible cells were counted but background was not. Cells identified by the program were visually assessed and false positives (marking fibers, bright areas of background and other aberrations) were manually removed. To assess proliferation of Ctip2+ and Satb2+ neurons, confocal images were cropped using ImageJ to a 400 μm-wide ROI spanning the cortical plate vertically. Within this ROI, total cells identified with Imaris as double positive for both BrdU and Ctip2, or for BrdU and Satb2 were divided by the total number of BrdU+ neurons in each image, to yield a ratio of proliferating cells bound for upper versus lower cortical layers.

#### Cell cycle kinetics

Cell cycle kinetics in E15.5 mice were assessed using Imaris software in combination with manual counting in an ROI (of variable size) incorporating the VZ and SVZ. The technique is described in detail in (48, 49). Briefly, the dam was injected with EdU at Time 0. Cells in S-phase at this time incorporate the EdU. Later, a second injection with BrdU is done and cells in S-phase at this time are marked. The cells remaining in S-phase at the time of dissection (S_cells_) are BrdU+, while cells that left the cycle prior to the BrdU injection (L_cells_) are EdU+/BrdU-. With T_i_ as the interval between the injections, the length of S-phase (T_s_) can be calculated (50) as follows: T_s_ = T_i_/(L_cells_/S_cells_). Then the length of the cell cycle (T_c_) can be estimated using the number of total proliferating cells, here labeled with Ki67 (P_cells_) as follows: T_c_ = T_s_/(S_cells_/P_cells_).

#### Assessment of cortical upper and deep layer principal cell densities

In P30 brains, a 750-μm-wide ROI containing the full vertical span of S1 cortex was assessed for number of Cux1+ and Foxp2+ cells. Cell densities were determined by using the ImageJ “Find Maxima” application on the clearest image in each z-stack. Tolerance was adjusted until visible cells were counted but background was not. The density of Cux1+ cells was calculated in layers II-IV of the S1 and the density of FoxP2+ cells was calculated in layers V/VI by dividing cell count by the area of the defined layers. Layer identity was determined based on DAPI counter-staining.

### Magnetic resonance imaging (MRI)

The procedures were the same as previously described and the current cortical data was obtained from the same set of brains as in (15).

#### Brain preparation

Anesthetized P30 mice were perfused intracardially with 30 mL of 0.1 M PBS containing 10 U/100 mL heparin (Sigma) and 2 mM ProHance (gadolinium contrast agent, Bracco Diagnostics, Monroe Township, NJ, USA), followed by 30 mL of 4% paraformaldehyde (PFA) and 2 mM ProHance. Following perfusion and decapitation, brains in skulls were postfixed in 4% PFA and 2 mM ProHance at 4°C overnight and then kept in PBS with 2 mM ProHance and 0.02% sodium azide for at least one month prior to MRI scanning (51, 52).

#### Diffusion tensor image (DTI) acquisition

Brain imaging was performed using a multi-channel 7 Tesla MRI scanner (Agilent Inc., Palo Alto, CA, USA). For DTI Imaging, brains were scanned with a 3D diffusion-weighted FSE scan (53, 54). The DTI sequence parameters were as follows: TR of 270 ms, echo train length of 6, intial TE of 32 ms, and a TE of 10 ms for the remaining 5 echoes, 1 average, FOV of 14 mm × 14 mm × 25 mm and a matrix size of 180 × 180 × 324 resulting in an image with 78 µm isotropic voxels. Using the Jones30 scheme (55), five b=0 s/mm^2^ images and 30 high b-value (b=2147 s/mm^2^) were acquired in 30 different directions. The entire imaging duration was about 12 hours. The FSL software package (FMRIB, Oxford, UK) was employed to generate fractional anisotropy and mean diffusivity (MD) maps for each brain sample.

#### MRI registration and analysis

The b=0 s/mm^2^ images were registered linearly (6 followed by 12 parameter) and non-linearly to determine any alterations to the mouse brains owing to genotype and sex. Registrations were carried out using a combination of mni_autoreg tools (56) and ANTS (advanced normalization tools) (57). After registration, all scans were resampled using an appropriate transform to create a population atlas depicting the average anatomy of the study samples. The final registration results in the individual images deformed into unbiased alignment with one another. Individual DTI parameter analyses compared intensity differences of MD between genotype and sex.

### Behavioral phenotyping

Animals underwent a series of behavioral tests beginning between 35 and 37 days of age, and completed the series within a two-week period. One test was carried out per day in the sequence described below at P35, P36 and P37 respectively.

#### Open field

Animals were habituated to the testing room for one hour, and then placed individually in the open field apparatus and recorded for 30 minutes. The open field was made up of a 16x16 square grid of 4 square inches. Mice were recorded from above with a USB webcam. Videos were analyzed with ANY-Maze software (Stoelting, Wood Dale, IL, USA). Average speed and total distance travelled during the trial were measured to assess overall activity level and motor function. Proportion of time spent in the central area (consisting of the central 4 squares in the grid) versus the surrounding squares was determined to assess anxiety and exploratory behavior. After testing, each animal was removed to a holding cage until all animals from the home cage had been tested. Between animals, the apparatus was wiped down with 70% ethanol.

#### Continuous spontaneous alternations Y-Maze

Spontaneous alternation is a simple, hippocampus-dependent task measuring working memory (58, 59). After habituation to the testing room for one hour in its home cage, the mouse was placed in the center of a Y-maze with arms labeled A, B and C, and allowed to freely explore. Arm choice was recorded by an observer blind to the genotype of the animal during an 8-minute period. The mouse was then removed to a holding cage (identical to the home cage) until all animals from the home cage had been tested to minimize disruption to animals yet to be tested. Between animals, the apparatus was cleaned with 70% ethanol. Spontaneous alternation behavior was scored by determining the number of three unique arm entries (alternation) divided by the total number of arm entries minus 1 (59).

#### Somatosensory novel object discrimination

This task is a modification of a standard novel object test (60) in which the animal is presented with two identical objects and then, following a period of time, with one of the original objects and a novel object. It tests recognition memory, a non-spatial memory paradigm. We ran this test under infrared light, to exclude the effects of vision and specifically test somatosensory system function. The initial objects were columns (2 inches high and 1 inch in diameter) made of hard plastic. The novel object was a large plastic binder clip, placed with metal prongs upright. This test was filmed from above and analyzed using ANY-Maze. Each animal was exposed to the initial objects under infrared light for 10 minutes on day 1 of testing. Following a 12-hour break, animals were exposed to one novel and one familiar object for ten minutes, with the side each was presented on systematically varied. After testing, each animal was removed to a holding cage until all animals from the home cage had been tested. Between animals, the apparatus was cleaned with 70% ethanol. Recognition Index (RI), which corresponds to the ratio between the time spent exploring the new object relative and the total exploration time, was assessed.

### Statistical analysis

Data analysis was performed using GraphPad Prism version 6.00 for Windows (GraphPad Software, www.graphpad.com). First, outliers over 2 standard deviations from the mean were excluded, and then normality of remaining data was assessed by the D’Agostino-Pearson normality test. For normal data, either 2-way ANOVA, followed by Sidak’s or unpaired Student’s t-test followed by Tukey *post-hoc* testing was used. Bonferroni corrections were used for multiple t-tests. For nonparametric data, we used the Mann-Whitney test. Expression data over time was analyzed with one-way ANOVA with tests for linear trend. Mean diffusivity (MD) data were analyzed for each cortical region using three-way ANOVA to assess the interaction effect between genotype, sex and region. Tukey’s method was used for multiple comparison *post-hoc* testing. Summary data are presented in the text as mean ± SEM from n animals. The p-values reflecting the likelihood of association between the experimental differentially expressed genes and diseases/functions given by Ingenuity Pathway Analysis were calculated using a right-tailed Fisher’s exact test. Differences were considered significant at p<0.05. Replicate numbers, p values and statistical tests used for each analysis are indicated in the figure legends. All measurements were performed in mice of both sexes and statistically analyzed by sex except where indicated.

## Results

### Cortical transcriptome is altered in adolescent mice following placental ALLO insufficiency

**Figure 1:**
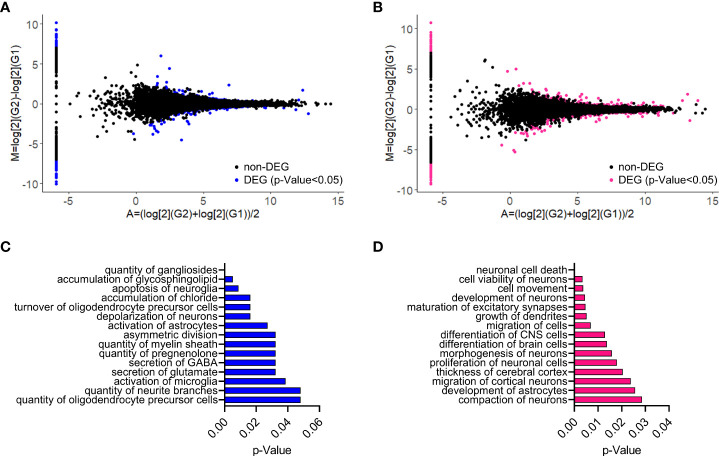
Impact of placental ALLO insufficiency on the cerebral cortex transcriptome at P30. **(A, B)** MA plots for the cortical RNAseq analysis of plKO vs Ctrl mice at P30 in males **(A)** and females **(B)**. M, log ratio; A, mean average. DEGs are represented as colored dots (blue for males and magenta for females). **(C, D)** Top 15 significant enriched gene functional groups identified with Ingenuity Pathway Analysis software (Qiagen) in males **(C)** and females **(D)**. P-values were calculated using right-tailed Fisher’s exact tests.

RNA-sequencing on whole cerebral cortex at P30 identified 162 upregulated and 169 downregulated genes in plKO males as compared with their littermate controls (**Figure 1A**; **Supplementary Figure 1A**; **Supplementary Table 2** sheet 1). In P30 females, we found 242 upregulated and 262 downregulated genes in plKO compared to control mice (**Figure 1B**; **Supplementary Figure 1B**; **Supplementary Table 2** sheet 2). Ingenuity Pathway Analysis of the significantly dysregulated genes showed significant disruption of myelin-related functions in plKO males (**Figure 1C**; **Supplementary Table 3A**), consistent with our previous findings on cerebellar white matter development alterations in plKO mice (15). Neural cell proliferation/survival/differentiation/migration pathway clusters also appeared among the top disrupted pathways in the plKO mice of both sexes, but in females more genes were altered (**Figure 1**; **Supplementary Tables 3**, **4**). Other commonly disrupted pathways involved neurophysiology, neuritogenesis and astrocytic functions (**Figures 1C, D**; **Supplementary Tables 3**, **4**). In the RNAseq analysis, *akr1c14* gene expression was unaltered in the P30 cerebral cortex (**Supplementary Table 2** sheets 1 and 2). Likewise, the levels of *akr1c14*-mRNA in the embryonic brain (at E14.5 and E17.5) and the postnatal cerebral cortex (from P0 to P15) were not different in plKO mice as compared with littermate controls (**Supplementary Figure 2**), suggesting that neither gene deletion nor regulation occurs in the plKO cortex, and thus ALLO production in the brain of plKO and control mice should be similar.

### Placental ALLO insufficiency causes long-term reduction of the mean diffusivity (MD) in specific cortical areas

**Figure 2:**
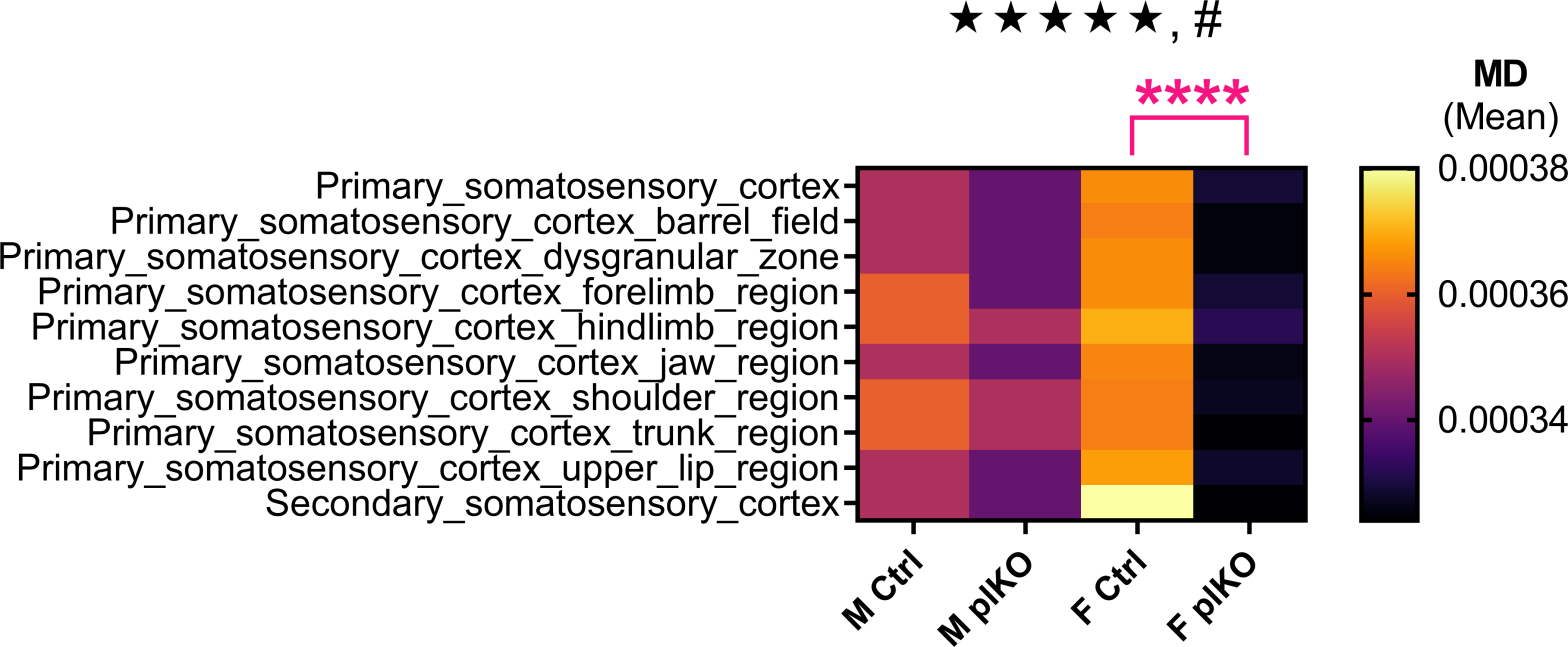
Diffusion Tensor Imaging (DTI) analysis in the somatosensory cortex of Ctrl and plKO mice at P30. Heatmap representing mean diffusivity (MD) across the different somatosensory areas. MD was significantly decreased in the female plKO somatosensory cortex, as compared with their littermate controls. Data presented in a hierarchical tree. Three-way sex by genotype by region ANOVA: main genotype effect: ★★★★★p<0.0005; sex × genotype interaction: #p<0.05. Tukey’s multiple comparison test: ****p<0.001. n = 11 M Ctrl, 8 F Ctrl, 13 M plKO, 11 F plKO. F, female; M, male.

Diffusion Tensor Imaging (DTI) was performed to investigate variations in cortical structural integrity throughout the different cortical areas. MD, which quantifies the rotationally invariant magnitude of water diffusion within the tissue, was compared between control and plKO mice at P30. Factorial ANOVAs were conducted to compare the main effects of genotype, sex and cortical area as well as their interactions on MD. In the somatosensory cortex, a significant effect of genotype on MD was found (F(1,390)=14.45, p=0.0002; **Figure 2**). There was also a significant interaction effect between genotype and sex (F(1,390)=4.727, p=0.0303). *Post-hoc* Tukey’s multiple comparison test revealed that MD was significantly lower in the female plKO than in female controls (p=0.0005). In the entorhinal areas, comparable, although less significant effects of genotype and sex × genotype interaction were seen (genotype: F(1,234)=6.095, p=0.0143; sex × genotype interaction: F(1,234)=6,987, p=0.0088; **Supplementary Figure 3A**). Multiple comparison tests found that MD was reduced in female plKO as compared with their controls (p=0.0043; Tukey’s test). Significant effects of genotype, without interaction with sex, was found in the cingulate (F(1,390)=10.31, p=0.0014; **Supplementary Figure 3B**) and orbital (F(1,156)=4.114, p=0.0442; 3-way ANOVA) cortices (**Supplementary Figure 3C**). In contrast, MD in frontal and motor areas was not significantly different in plKO as compared with control mice (not shown). Collectively, these results indicate that placental ALLO loss is associated long-term microstructural changes of the cerebral cortex, with females being particularly vulnerable in areas such as the somatosensory cortex.

### The density of upper-layer S1 neurons is durably altered in plKO female mice

**Figure 3:**
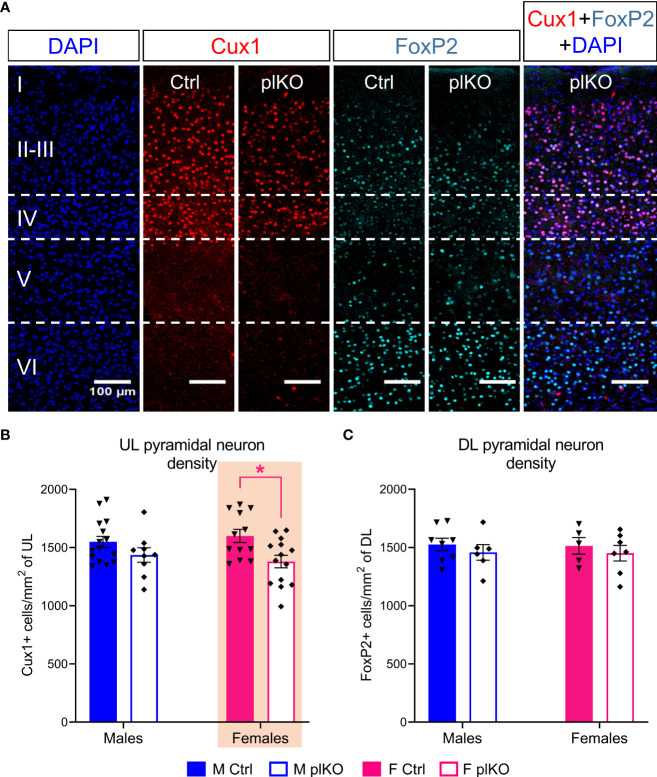
Long-term effect of placental ALLO insufficiency on cortical lamination. **(A)** Representative immunofluorescent staining for Cux1 (red) and FoxP2 (cyan) in the female S1 area at P30. **(B, C)** Quantification of **(A)** shows that the density of Cux1+ upper layer principal cells is significantly decreased (layers II-IV) in plKO females but that the density of FoxP2+ deeper layer principal cells remained unaltered. n = 8-15 M Ctrl, 5-12 F Ctrl, 6-9 M plKO, 7-14 F plKO. Data presented as mean ± SEM. Two-way sex by genotype ANOVA with Sidak’s *post-hoc* comparison test: *p < 0.05 identified in magenta for females. Cux1, Cut like homeobox 1; F, female; FoxP2, Forkhead box protein P2; M, male. DL, deep layer; UL, upper layer.

Based on our DTI characterization of the plKO brain which identified sex-linked MD changes in the somatosensory cortex, we next examined the cyto-architectonic organization of the primary somatosensory cortex (S1). Principal neuron populations were assessed using the post-mitotic layer markers Cux1 and FoxP2, which label UL and DL pyramidal neurons, respectively. A significant reduction of Cux1+ neurons in the UL S1 was seen in P30 plKO females as compared with littermate controls, but not in males (Males: p=0.32; Females: p=0.01; Sidak’s multiple comparison test; **Figures 3A, B**). In contrast, there was no significant difference in the density of DL FoxP2+ cells by genotype or sex (**Figures 3A, C**). This data shows that the lack of placental ALLO alters the S1 UL neurons in a sex- and layer-dependent manner.

### Long-term, female-specific behavioral impact of placental ALLO insufficiency

**Figure 4:**
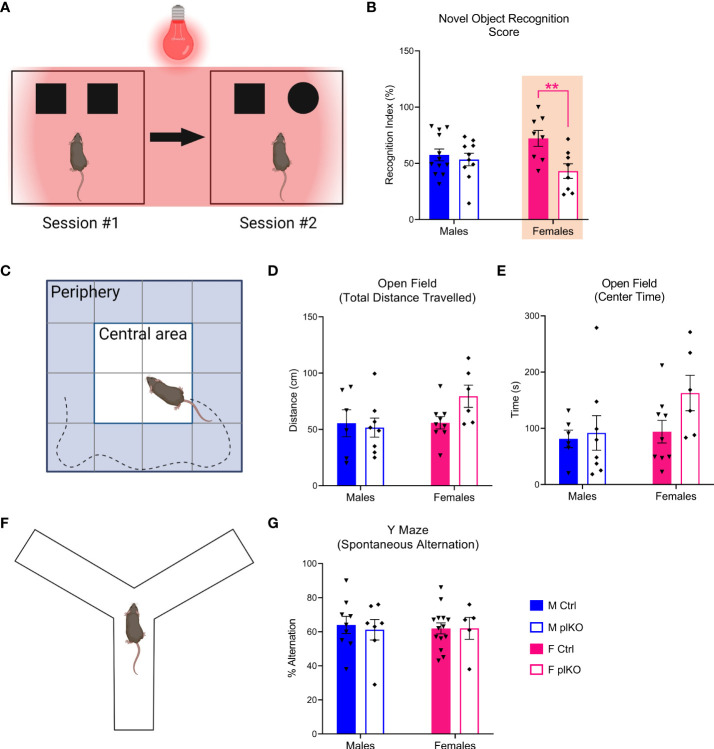
Long-term effect of placental ALLO insufficiency on behavior. **(A)** Schematic diagram of the novel object recognition test, designed to assess the somatosensory function. **(B)** Female plKO mice have significant deficits in the novel object recognition task, as measured by the recognition index (RI), which corresponds to the ratio between the time spent exploring the new object relative and the total exploration time. n = 12 M Ctrl, 8 F Ctrl, 10 M plKO, 8 F plKO. Data presented as mean ± SEM. **(C)** Diagram of open field paradigm. **(D)** Total distance travelled by Ctrl and plKO mice in open field test. **(E)** Time spent in center quadrants of the open field. n = 6 M Ctrl, 9 F Ctrl, 8 M plKO, 6 F plKO. **(F)** Diagram of alternating Y-maze. **(G)** Alternation score (# alternations/# of possible alternations x 100). n = 9 M Ctrl, 14 F Ctrl, 7 M plKO, 5 F plKO. Data presented as mean ± SEM. Two-way sex by genotype ANOVA with Sidak’s *post-hoc* comparison test: **p < 0.01 identified in magenta for females. Illustrations in **(A, C, F)** were prepared using BioRender.com.

To assess the somatosensory function of plKO mice, P30 mice underwent a modified novel object recognition (NOR) test. The NOR task was performed under infrared light to exclude the use of visual cues (**Figure 4A**). Recognition Index, the ratio of the time spent exploring the new object relative to the total exploration time, was assessed. The Recognition Index was significantly decreased in plKO females but not in males, as compared with sex-matched littermate controls (Males: p=0.84; Females: p=0.007; Sidak’s multiple comparison test; **Figure 4B**), suggesting that plKO females were less able to discriminate between the known and novel objects due to altered somatosensory processing, potentially linked to the anatomical changes noted in S1. PlKO mice, however, did not differ from controls in tests of activity (open field) (**Figures 4C–E**) and spatial memory (Y-maze) (**Figures 4F, G**).

Additional physiological and behavioral assessments included growth and reproduction function. Body weight was monitored across postnatal development until adulthood, but was not influenced by genotype (**Supplementary Figure 4**). As previously reported, both mothering and mating behaviors appeared unaltered (15).

### Placental ALLO insufficiency disrupts proliferation dynamics in the S1

GABA signaling has a strong trophic effect in the developing cortex (19) and ALLO, as a GABAergic modulator, has been implicated in the regulation of neural progenitor cell proliferation and neurogenesis (21, 22, 61, 62), so embryonic neurogenesis was assessed in the S1 in embryos with placental ALLO loss compared to normal placental exposure. Insufficiency in placental ALLO in plKO mice is detected as early as E14.5 and persists until at least E17.5 (15), which corresponds to a critical window for UL neurogenesis in the mouse cerebral cortex.

**Figure 5:**
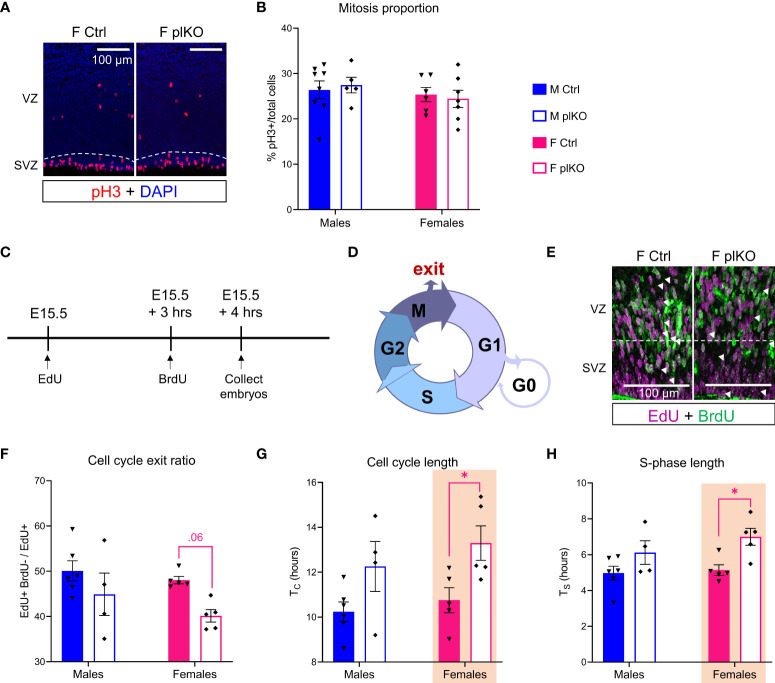
Influence of placental ALLO insufficiency on mitosis and cell cycle kinetics in the cerebral cortex during mid-to-late gestation. **(A)** Immunofluorescent staining of pH3, a mitotic marker, in the cerebral cortex at E15.5. **(B)** Quantification of **(A)** reveals no change in the proportion of SVZ cells undergoing active mitosis in the plKO cerebral cortex at E.15.5. n = 8 M Ctrl, 6 F Ctrl, 5 M plKO, 7 F plKO. **(C)** Overview of experiments to determine cell cycle exit and kinetics at E15.5. **(D)** The different phases of cell cycle. **(E)** Immunofluorescent staining of EdU (magenta), marking proliferating cells at E15.5, dual pulsed with BrdU (green), injected 3 hours later, to identify cells in cycle and cells that had exited. **(F)** Quantification of the cell cycle exit ratio in the SVZ shows a significant loss in cell cycle exit in plKO mice at E15.5. **(G)** Significant increase in the SVZ cell cycle length (Tc) in plKO females associated with an **(H)** elongated S-phase (T_S_). n = 6 M Ctrl, 5 F Ctrl, 4 M plKO, 5 F plKO. Data presented as mean ± SEM. Two-way sex by genotype ANOVA with Sidak’s *post-hoc* comparison test: *p < 0.05 identified in magenta for females. F, female; M, male; pH3, phospho-Histone 3; SVZ, subventricular zone.

We first examined cell proliferation in the SVZ at E15.5, when ALLO insufficiency in plKO mice is the greatest and UL corticogenesis begins. Mitosis, assessed by phospho-Histone 3 (pH3) immunostaining, was similar in controls and plKOs at E15.5 (Males: p=0.91; Females: p=0.92; Sidak’s multiple comparison test; **Figures 5A, B**). To assess whether the lack of placental ALLO was associated with increased apoptosis, we tested the E15.5 cortex for the ratio of the pro-apoptotic protein BAX and the anti-apoptotic protein BCL2 in females (63, 64). There was no significant change in this ratio (**Supplementary Figure 5**), indicating normal levels of apoptosis in plKO S1.

Since no change in cell proliferation or death was found, we next asked whether cell cycle exit and kinetics were altered by reduced placental ALLO exposure. Dual pulse labeling experiments were carried out as follows: EdU was injected at E15.5 and BrdU 3 hours later, and embryos were then collected one hour after the last injection (**Figures 5C, D**). Cell cycle exit ratio, defined by the ratio of (EdU+BrdU-)/EdU+, was reduced in female plKOs compared to controls in the SVZ, although not reaching statistical significance (Males: p=0.28; Females: p=0.06; Sidak’s multiple comparison test; **Figures 5E, F**). Cells had significantly longer cell cycles (T_C_) in female plKOs compared to controls (Males: p=0.12; Females: p=0.03; Sidak’s multiple comparison test; **Figure 5G**), due to significantly longer S-phase (T_S_; Males: p=0.18; Females: p=0.02; Sidak's multiple comparison test; **Figure 5H**). Regulation of the S-phase is typically the main driver of cell cycle length (65). However, T_S_ was only 90 minutes longer in plKO females as compared with controls, and cannot fully explain the 2½ hour increase of T_C_, suggesting that other phases of cell cycle are also elongated.

### Placental ALLO insufficiency impairs the production of UL neurons in the S1

**Figure 6:**
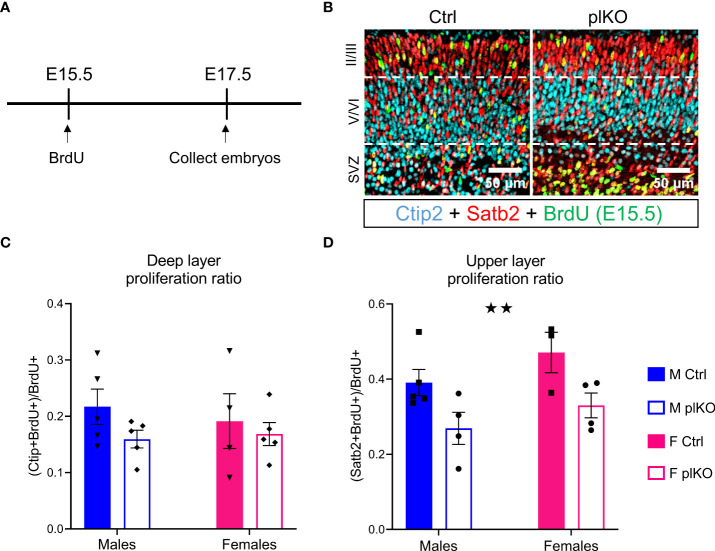
Effect of placental ALLO insufficiency on cortical neurogenesis. **(A)** Overview of the experiments: BrdU was injected at E15.5, and embryos were euthanized at E17.5. **(B)** Determination of the localization of proliferating E15.5 cells (BrdU, green) by co-staining with Satb2 (red) upper layer principal cell marker and Ctip2 (cyan) lower layer principal cell marker. **(C)** Quantification of **(B)** shows that while deeper layers were unaltered, **(D)** fewer E15.5 born cells localized to the upper layers of the cortical plate. n = 5 M Ctrl, 4-5 F Ctrl, 3-4 M plKO, 4-5 F plKO. Data presented as mean ± SEM. Two-way sex by genotype ANOVA with Sidak’s *post-hoc* comparison test. Main effect of genotype: ★★p < 0.01. Ctip2, COUP-TF-interacting protein 2; F, female; M, male; Satb2, Special AT-rich sequence-binding protein 2. SVZ, sub-ventricular zone.

To investigate the consequences of altered cell cycles on the production of cortical plate neurons, we next assessed the production of E15.5 born neurons at E17.5 (marked by BrdU; **Figure 6A**). The layer markers Ctip2 and Satb2 were used to identify early-born, DL-bound principal cells and late-born, UL-bound principal cells, respectively (66–68). There was no difference in the ratio of Ctip2+ BrdU+ cells relative to total BrdU+ cells, indicating that the E15.5 born neurons destined for deep layers were produced and migrated normally (**Figures 6B, C**). In contrast, between E15.5 and E17.5, 30% fewer proliferating E15.5 cells became UL, Satb2+ neurons in the plKO S1 compared to controls (p=0.0072; 2-way ANOVA; **Figures 6B, D**), showing that placental ALLO insufficiency primarily affects UL cortical development in the embryonic period.

### Cortical layering tends to be altered in female preterm infants

We investigated whether human preterm birth, which results in acute, early loss of placental endocrine support, was also associated with altered cortical lamination. We examined 22 post-mortem specimens comprising Brodmann Area 10 from term (>37 weeks of gestation) and preterm (<34 weeks of gestation) infants that died at an average corrected age of 6 weeks (**Supplementary Figure 6A**). We found that the ratio of *cux1*-mRNA to *foxP2*-mRNA levels tend to be decreased in the preterm females but not the males (Males: p=0.96; Females: p=0.09; Sidak’s multiple comparison test; **Supplementary Figure 6B**). The female-specific trend toward reduction of UL/DL neuronal ratio parallels our rodent findings, although significance at p<0.05 was not reached, possibly due to the small sample size available.

## Discussion

Children born prematurely are at higher risk for developing cognitive difficulties in areas such as perceptual-motor function, learning and memory, attention, and social cognition. Since the placenta is lost prematurely in all preterm births, we have hypothesized that absence of late gestation placental factors could be a contributing factor. We have specifically investigated the role of placental ALLO in neurodevelopment because this neurosteroid is primarily produced by the placenta during late gestation, crosses the blood brain barrier, and has known neurotrophic properties. We created and validated a placenta-specific KO mouse line (plKO), in which placental ALLO production and resulting placental and brain ALLO levels are reduced (15). Here we show that placental ALLO insufficiency impairs cortical development at cellular and functional levels, with greater and persistent alterations in female cortex, and female neurosensory behavioral changes. These findings contrast with our previously reported findings of greater male susceptibility to cerebellar myelination changes with an ASD-like phenotype (15). Susceptibility to white matter decrements in males and cortical grey matter loss in females born preterm has been reported (69). Together these findings emphasize the need to examine sex, region and cell type specificity when examining the neurological impact of placental dysfunction.

Our unbiased approach to examining postnatal plKO cortex at multiple scales, from transcriptomics to MRI, revealed that placental ALLO loss is associated with long-term, sex-linked molecular and structural alterations. RNA sequencing analyses of the total cerebral cortex indicated greater dysregulation of neuronal and myelin development-related pathways in females and males, respectively, although there were many shared alterations on other pathways as well.

The current transcriptomic analysis suggests that white matter alterations occur not only in the cerebellum (15) but also across the brain in male plKOs. Although brain myelination is primarily a postnatal process, oligodendrocyte lineage cells are mostly produced embryonically (70), when placental ALLO is highest. Previous studies have shown the critical role played by ALLO in the development and maturation of oligodendrocyte lineage cells through GABA_A_-R signaling (71). It is not surprising, then, that ALLO insufficiency during fetal life disrupts brain myelination in plKO mice. A full characterization of the plKO white matter tracts is now needed to determine whether myelination impairments are consistent across brain regions, and how sex influences myelination trajectories.

Female mice lacking placental ALLO exhibit impaired cell cycling in the SVZ at E15.5, consistent with ALLO’s known actions on neurogenesis (22, 67, 72). Additional downstream modulators of neurogenesis, such as BDNF or IGF-1, both regulated by ALLO (73, 74), may also contribute to altered neurogenesis in response to ALLO deficits. ALLO is known to promote neurogenesis by upregulating genes that shorten cell cycle length (22) *via* GABA mediated downstream calcium signaling (75), it is thus likely that the mechanism underlying plKO alterations is also GABAergic. During early development, GABA has a potent trophic effect in the cerebral cortex (19). When gestational ALLO is high, GABA_A_R-mediated responses depolarize progenitor cells and immature neurons due to high intracellular chloride concentration (31, 76), increasing excitability and stimulating spiking and Ca^2+^ entry. Calcium entry can then activate a variety of signaling cascades controlling cell proliferation, migration, neuritogenesis, and synaptogenesis (77). The lengthened cell cycles in the SVZ of plKO females may reflect a delayed cortical maturation because cell cycle length normally decreases over time (78). Prolonged cell cycle may also indicate that IPCs undergo quiescence, which can persist for at least 48 hours (79). Ultimately, cells in S-phase will undergo mitosis, with cell cycle length determining the fate of divisions. Shorter cell cycle length is associated with a pro-differentiating progenitor state (80), while prolonged cell cycle shift IPCs toward self-renewal (75, 81). Further investigation is needed to determine whether IPCs in plKOs are shifted toward proliferation; such a change would result in a loss of differentiated UL neurons, as seen in plKO mice.

A major feature of the mammalian neocortex is its laminar organization, with each layer displaying distinct connectivity and function (35). Reduced cortical thickness in multiple areas, including the S1, is common in human preterm survivors (82–85), but specific layer alterations have not been elucidated. In plKO mice, the density of S1 UL neurons born at E15.5 was decreased at P30 in females. However, no evidence of resultant increased DL cell production or density, nor apoptosis, was found. This difference may lie in the timing of DL versus UL cell production. The formation of the cortical DL and UL takes place at E11.5-E14.5 and E13.5-E16.5, respectively (86). In control placentae, the expression of *akr1c14* peaks at E14.5, which coincides with the most pronounced ALLO insufficiency in plKO mice (15). Thus, in our model, the impact of placental ALLO insufficiency on corticogenesis is expected to peak at E14.5, a stage at which the production of cortical DL neurons is nearly complete, while the UL population is still actively expanding. In plKO mice, the UL-specific alteration was seen as early as E17.5 with a reduction of the layer II/III Satb2+ cells born at E15.5 in both sexes. This decrease persisted at P30 with a deficit in Cux1+ neurons in the females. Cortical layer specification underlies specific patterns of axonal projections. Satb2 and Cux1 both enable the specification of callosal projection neurons (68, 87). In the human cortex, Cux1+ UL cells are still being produced at gestational week 23, near the lower limit for premature survival, and migrate until gestational week 37 (88). Lack of placental ALLO upon premature birth in infants might interrupt proliferation and migration of the future callosal neurons. Our molecular analysis of cortical lamination in preterm infants lends some initial support to this hypothesis since the ratio of the UL marker Cux1 on the DL marker FoxP2 tends to decrease in females.

Ultimately, loss of UL principal neurons has wide-ranging effects on S1 function because of the hierarchical nature of somatosensory information processing. Thalamo-cortical projections target layer IV neurons, which innervate layer II/III cells that feed-forward onto layer V for integration and output to other cortical regions (89). In mice, barrels of layer IV are somatotopically mapped for individual whisker (90, 91) while layer II/III plays an essential role in regulating layer V excitation during tactile whisker response (92). In plKO females, the UL cortical cell deficit in the S1 could underlie significant impairment in the whisker-specific, novel object recognition task. Alternative explanations for this specific deficit include decreased preference for novelty or altered memory, rather than a somatosensory deficit, but the plKO mouse showed no avoidance for novelty, spending equivalent time in the center of the open field test as control mice, and investigated arms of the Y-maze with the same frequency. Thus, somatosensory-specific functioning is impaired following placental ALLO insufficiency, correlating with the observed anatomical changes. Survivors of premature birth often have functional deficits in somatosensory processing too (93), which impair their social behavior, motor skills, and ability to complete tasks of daily living (94–97). Additionally, reduced connectivity (altered UL, callosal neurons) associated with Attention Deficit Disorder (98) or Autism Spectrum Disorders (ASD) (99) are commonly seen in premature infants (100–102). The extent of these behavioral impairments in preterm survivors is associated with cortical thickness (83) as well as with sex-dependent effects in somatosensory processing (93). Our findings bolster previous theories that loss of neurons in the S1 underlies deficits in somatosensory activity in preterm infants (103).

Our DTI analysis showed reduced MD in the somatosensory cortex of female plKOs, suggesting long-term, area- and sex-linked changes in cortical microstructure. However, the precise nature of the histological substrate underlying this altered DTI remains unclear. Given our transcriptomic and cellular analyses, we would expect that neuronal density is a substantial contributing element. However, prior studies comparing histology and neuroimaging data have shown that MD can correlate positively with neuronal density, at least in the hippocampus (104), complicating this interpretation of MD. Based on our RNAseq Ingenuity Pathway Analysis results, other contributing factors could include altered neurite and radial glia fiber development, which will be investigated in the future. Additionally, although our DTI analysis of the plKO cortex suggests altered microstructural integrity in the female S1, it also indicates that such anomaly may not be limited to the S1, but may affect other cortical areas such as the entorhinal cortex. Broader investigation of additional cortical functions is now required to decipher the global impact of placental ALLO insufficiency on the development of cognitive functions.

A striking sexual dimorphism was seen in the S1 development of mice lacking placental ALLO. In plKO mice, deficits in neurogenesis, cortical lamination and somatosensory function are more significant and long-lasting in the female than in the male progeny, revealing an increased female cortical vulnerability to placental ALLO insufficiency. Although placental ALLO levels and brain *akr1c14* gene expression are similar between sexes, an accentuation of sexually distinct features seems to take place in the plKO brain (15). For instance, a marked sex-divergence in the myelination rate was seen in the plKO cerebellum (15). In several brain regions, females express fewer GABA_A_-R subunits and less glutamic acid decarboxylase (105, 106), and GABA has been proposed as a pivotal factor in the divergence of male vs female neurodevelopment (107). In particular, the capacity of GABA to change neuronal excitability in a depolarizing rather than hyperpolarizing manner may have major implications for a neuron’s phenotype, activating diverse signal transduction pathways and eventually resulting in varied gene expression. Sex-linked differences in receptor and transporter distribution or maturational state may contribute to sex-linked differences in response to GABA or its loss. For instance, in mice, the female brain is more sensitive to ALLO-mediated GABAergic neurotransmission potentiation, which contributes to enhanced neuroprotection from ischemia (108). Whether cortical GABAergic signaling is altered in plKO mice and contributes to the sex differences in their cortical phenotype is an area of active investigation.

The lack of placental ALLO may represent a contributing factor to various neurological disorders following prematurity or impaired placental function, with temporal and sex-linked effects on corticogenesis and somatosensory function. Our findings in mouse and human cortex further suggest that loss of placental ALLO could contribute to the sexually dimorphic outcomes of preterm birth. The DTI and RNAseq analyses of the plKO brain also indicate that placental ALLO insufficiency may alter broader cellular processes that vary by brain regions. Further investigation of the plKO brain development will provide valuable knowledge for future development of therapeutic strategies aimed at preventing long-term neurodevelopmental disorders that result from loss of placental function.

## Data availability statement

The datasets presented in this study can be found in online repositories. The names of the repository/repositories and accession number(s) can be found below: https://www.ncbi.nlm.nih.gov/, GSE173440.

## Ethics statement

Ethical review and approval was not required for the study on human participants in accordance with the local legislation and institutional requirements. Written informed consent for participation was not required for this study in accordance with the national legislation and the institutional requirements. The animal study was reviewed and approved by Children’s National Research Institute, Washington, DC 20010 USA.

## Author contributions

DB, JO’R, C-MV, and AP designed research. DB, JO’R, HL, JS, JE, YI, and C-MV performed research. DB, JO’R, JE, JL, TS, YI, KH-T, C-MV, and AP analyzed data. DB, JO’R, C-MV, and AP wrote the paper. All authors contributed to the article and approved the submitted version.

## Funding

This work was funded by National Institutes of Health grants R01HD092593 and 3R01HD092593-S1 (AP) and F31HD098886 (JO’R), the DC-IDDRC U54HD090257 (PI: Gallo), the Children’s National Board of Visitors (AP) and the Research Foundation of Cerebral Palsy Alliance (no. 3720; AP).

## Acknowledgments

We thank Dr. G. Leone for providing us with the first Cyp19a-Cre mouse breeders. We also would like to express our gratitude to the families for their donation to the NIH NeuroBioBank, as well as to J. Cottrell (NIH NeuroBioBank), who assisted us with tissue selection. We thank William Yakah for his help with data presentation. We thank the Children’s Research Animal Facility, directed by J. Bradford, for taking care of our mouse colonies. We also acknowledge the support of the DC-IDDRC headed by Dr. Vittorio Gallo. In particular, we would like to thank the DC-IDDRC Animal Neurobehavioral Core, led by Dr. J. Corbin, for providing advice, space and equipment to conduct the behavioral phenotyping of our mice, and the DC-IDDRC Cell and Tissue Microscopy Core, headed by Dr. Jyoti Jaiswal, for providing access to light and confocal microscopes and image processing software. We would also like to acknowledge the Ontario Brain Institute (OBI) for financial support (JE and JL).

## Conflict of interest

The authors declare that the research was conducted in the absence of any commercial or financial relationships that could be construed as a potential conflict of interest.

## Publisher’s note

All claims expressed in this article are solely those of the authors and do not necessarily represent those of their affiliated organizations, or those of the publisher, the editors and the reviewers. Any product that may be evaluated in this article, or claim that may be made by its manufacturer, is not guaranteed or endorsed by the publisher.

## Supplementary material

Supplementary Figure 1 Impact of placental ALLO insufficiency on the cerebral cortex transcriptome at P30. **(A, B)** Heatmaps of the significant differentially expressed genes at P30 in Ctrl and plKO samples, in males **(A)** and females **(B)**. Genes and samples were hierarchically clustered based on Pearson distance of z-score data and average linkage. n=3 independent samples/group.

Supplementary Figure 2 Time-course changes in *akr1c14* gene expression in the embryonic brain and postnatal S1 somatosensory cortex. *Akr1c14*-mRNA level fold changes to E14.5 male control mean, determined by qRT-PCR and after normalization to *ywhaz*. Data presented as mean ± SEM. F, females; M, males; ywhaz, Tyrosine 3-Monooxygenase/Tryptophan 5-Monooxygenase Activation Protein Zeta. Data presented as mean fold change ± SEM. Two-way age by genotype and sex ANOVA with Sidak’s *post-hoc* comparison test. Main effect of age: p<0.0001.

Supplementary Figure 3 Diffusion Tensor Imaging (DTI) analysis by cortical area in Ctrl and plKO mice at P30. Heatmaps representing mean diffusivity (MD) throughout the different cortical areas. **(A)** Significant effects of genotype and sex × genotype interaction were found in the entorhinal cortex (3-way ANOVA). MD was significantly decreased in the female plKO as compared with littermate controls (Tukey’s test). A main genotype effect, with no significant interaction with sex, was seen in the cingulate **(B)** and orbital **(C)** cortices. Three-way sex by genotype by region ANOVA: main genotype effect: ★p<0.05, ★★★p<0.005; sex × genotype interaction: ##p<0.01. Tukey’s multiple comparison test: ***p<0.005. n=11 M Ctrl, 8 F Ctrl, 13 M plKO, 11 F plKO. F, female; M, male.

Supplementary Figure 4 Body weight in the Ctrl and plKO males **(A)** and females **(B)** across early postnatal development and adulthood. Data presented as mean ± SEM. Two-way age by genotype repeated measures ANOVA with Sidak’s *post-hoc* comparison test. Main effect of age in males and females: p<0.0001.

Supplementary Figure 5 Apoptosis rate in the plKO cerebral cortex at E15.5. **(A)** Western blot analysis of the pro-apoptotic protein BAX and the anti-apoptotic protein BCL2. **(B)** Quantification showing that there is no change in the BAX/BCL2 ratio relative to β-tubulin in plKO female mice as compared with controls. n=5 Ctrl and 6 plKO. Data presented as mean ± SEM. Mann-Whitney test. BAX, BCL2 Associated X protein; BCL2, B-cell lymphoma-2 protein.

Supplementary Figure 6 Preterm birth is associated with dysregulation of layer-specific marker expression in the Brodmann Area 10. **(A)** Schematic of the age terminology during the perinatal period used for the human study. **(B)** Cux1-mRNA/FoxP2-mRNA ratio determined by qRT-PCR. n=9 male term, 4 female term, 4 male preterm, 5 female preterm. Data is presented as mean fold-changes ± SEM. Two-way sex by term ANOVA with Sidak’s *post-hoc* comparison test. (A) was created with BioRender.com.

Supplementary Table 1 Sequences of the PCR primers.

Supplementary Table 2 List of the significantly dysregulated genes in the cerebral cortex of P30 plKO males (sheet 1) and females (sheet 2), as compared with their littermate controls.

Supplementary Table 3
**(A, B)** Top 15 significant enriched pathways in plKO cerebral cortex with p-Values and associated genes. Enriched gene functional groups identified with Ingenuity Pathway Analysis software (Qiagen) in males **(A)** and in females **(B)**.
